# Factors Influencing Neurodevelopment after Cardiac Surgery during Infancy

**DOI:** 10.3389/fped.2016.00137

**Published:** 2016-12-15

**Authors:** Hedwig Hubertine Hövels-Gürich

**Affiliations:** ^1^Pediatric Cardiology, University Hospital RWTH Aachen, Aachen, Germany

**Keywords:** risk factors, neurodevelopment, congenital heart disease, cardiac surgical procedures, cardiopulmonary bypass, brain MRI, encephalopathy

## Abstract

Short- and long-term neurodevelopmental (ND) disabilities with negative impact on psychosocial and academic performance, quality of life, and independence in adulthood are known to be the most common sequelae for surviving children after surgery for congenital heart disease (CHD). This article reviews influences and risk factors for ND impairment. For a long time, the search for independent risk factors was focused on the perioperative period and *modalities of cardiopulmonary bypass (CPB)*. CPB operations to ensure intraoperative vital organ perfusion and oxygen supply with or without circulatory arrest or regional cerebral perfusion bear specific risks. Examples of such risks are embolization, deep hypothermia, flow rate, hemodilution, blood gas management, postoperative hyperthermia, systemic inflammatory response, and capillary leak syndrome. However, influences of these *procedure-specific risk factors* on ND outcome have not been found as strong as expected. Furthermore, modifications have not been found to support the effectiveness of the currently used neuroprotective strategies. *Postoperative factors*, such as need for extracorporal membrane oxygenation or assist device support and duration of hospital stay, significantly influence ND parameters. On the other hand, the so-called “innate,” less modifiable *patient-specific risk factors* have been found to exert significant influences on ND outcomes. Examples are type and severity of CHD, genetic or syndromic abnormalities, as well as prematurity and low birth weight. Structural and hemodynamic characteristics of different CHDs are assumed to result in impaired brain growth and delayed maturation with respect to the white matter. Beginning in the fetal period, this so-called *“encephalopathy of CHD”* is suggested a major innate risk factor for pre-, peri-, and postoperative additional hypoxic or ischemic brain injury and subsequent ND impairment. Furthermore, MRI studies on brain volume, structure, and function in adolescents have been found correlated with cognitive, motor, and executive dysfunctions. Finally, *family and environmental factors* independently moderate against ND outcomes. In conclusion, the different mediating factors may exert independent effects on ND and interactive influences. Implications for the future comprise modifying clinical risk factors, such as perioperative cerebral oxygen delivery, conducting brain MRI studies in correlation to ND outcomes, and extending psychosocial interventions leading to adequate resilience.

## Introduction

The prevalence of congenital heart disease (CHD) is about 1 in every 100 live births. About one-third of CHD cases is in critical need of surgical intervention in neonatal or infant age (1). Since the 1980s, advanced diagnostic technologies, neonatal cardiopulmonary bypass (CPB) operations enabling early correction of complex congenital heart defects, and improved postoperative care have markedly increased life expectancy: today more than 90% of CHD patients survive into adulthood. At the same time, these patients are at remarkable risk of short- and long-term neurodevelopmental (ND) impairment. This can have negative impact on psychosocial and academic performance, quality of life, and independence in adulthood (2–6).

The present article discusses causes and factors mediating risk for ND disabilities from neonatal to adolescent age in CHD patients after cardiac surgery during infancy.

## Influencing Factors on Neurodevelopment: Innate and Modifiable Parameters

**Figure 1:**
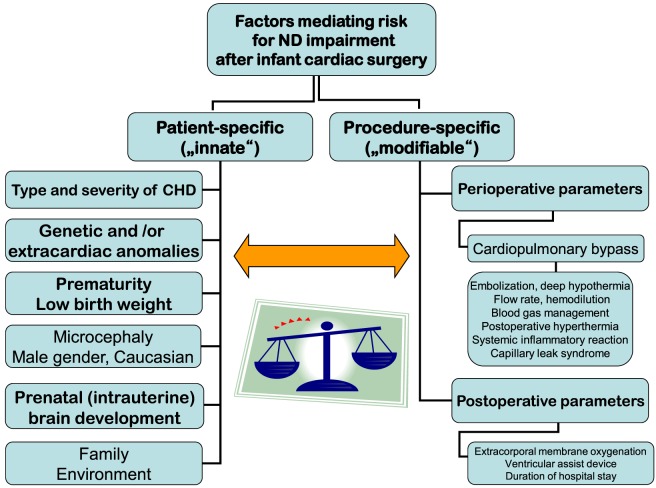
**Factors-mediating risk for neurodevelopmental impairment after infant cardiac surgery**.

Risk factors for brain injury and consecutive ND disabilities in infants, children, and adolescents with CHD after cardiac surgery in infancy may exert independent, cumulative, and synergistic influences. They comprise patient-specific (mostly innate and not modifiable) and procedure-specific (in part modifiable) parameters (Figure 1).

For a long time, the search for independent factors mediating risk for ND impairment has focused on the perioperative period and modalities of CPB. CPB operations to ensure intraoperative vital organ perfusion and oxygen supply with or without circulatory arrest or regional cerebral perfusion bear specific risks. Examples for such risks are embolization, deep hypothermia, flow rate, hemodilution, blood gas management, postoperative hyperthermia, systemic inflammatory response, and capillary leak syndrome (7–11). Though numerous modifications of these factors have been performed over time, ND outcomes have not improved accordingly (12, 13). In a recent analysis of >1,700 CHD patients from across the world who were born between 1996 and 2009 and had cardiac surgery at age <9 months, only modest improvements in the significantly reduced ND outcomes (psychomotor developmental index—PDI and mental developmental index—MDI) of the Bayley Scales of Infant Development-II at a mean age 14 months have been observed (14). Moreover, CPB management factors explained only about 1% of test results’ variance. Longer support time was hypothesized to be a surrogate for operative complexity. Postoperative parameters like need for extracorporal membrane oxygenation or ventricular assist device support and longer postoperative length of hospital stay were associated with lower ND results. In total, measured intraoperative and postoperative factors accounted for 5% of the variances in PDI and MDI (15).

There is a strong evidence that less modifiable innate patient characteristics and socioeconomic environmental factors have an important impact on ND outcomes (14, 16–19). In addition to type and severity of CHD, prematurity, lower birth weight, white race, and genetic or extracardiac anomalies have been assessed as predicting lower PDI. Lower birth weight, male gender, less maternal education, and genetic or extracardiac anomalies were independent risk factors for lower MDI (14). In general, genetic disorders are found in about 30% of patients with CHD. This includes chromosomal disorders, microdeletions, or mutations. However, only about one-third of the variance in ND outcomes early after cardiac surgery in infancy can be explained by the known innate patient and preoperative risk factors. Further genetic and epigenetic factors (changes in proteins affecting gene regulation) (20) or genetic polymorphisms such as the apolipoprotein E affecting the resilience capability of the brain (18) may exert influences on ND outcomes.

In addition, psychosocial factors comprising family and environmental parameters moderate against, or augment adverse ND behavioral and school outcomes. Family factors comprise parenting style such as overprotection, maternal mental health, and worry. They are able to exert significant influence on cognitive outcomes. Socioeconomic status is considered the most important environmental factor (21, 22).

## Encephalopathy of CHD: Delayed Maturation, Injury, and Neurodevelopment

There is evidence that structural and hemodynamic characteristics of different congenital heart defects lead to autoregulation mechanisms in the brain in case of hypoperfusion or hypoxia by vasodilatation of the cerebral arteries with increased diastolic flow and decreased cerebrovascular resistance (23, 24). It has been assumed that prolonged periods of this autoregulation may lead to delayed maturation of the fetal oligodendrocytes, reduced myelinization, and increased vulnerability of the brain (25–28). However, in fetuses with single ventricular heart, decreased cerebrovascular resistance has been found associated with higher PDI scores at the age of 14 months (29, 30). It remains unclear whether fetal cerebral blood flow alterations predict ND outcomes later in childhood.

Fetal brain perfusion disturbance has been supposed to result in impaired brain growth and maturation with respect to the white matter. Neuropathological studies indicate that the brain disturbance of infants with CHD consists predominantly of cerebral white matter injury (WMI). This result is comparable to periventricular leucomalacia as described in preterm infants (31, 32).

**Figure 2:**
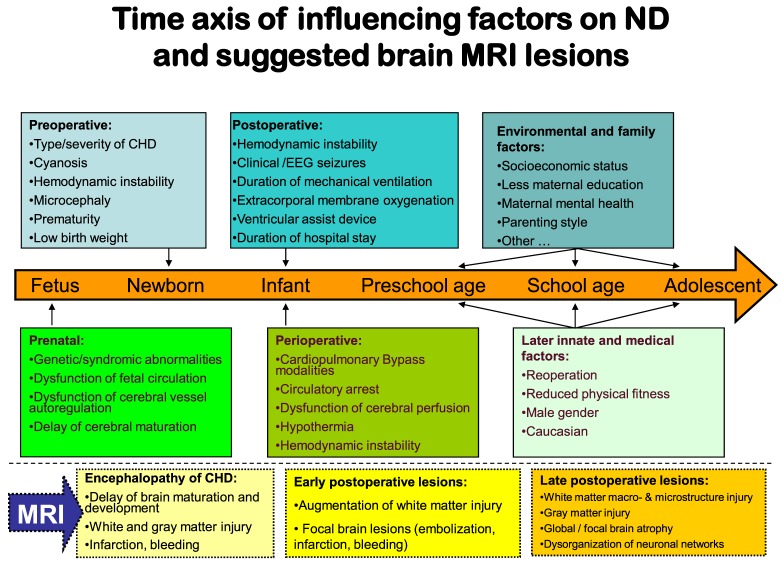
**Factors-influencing neurodevelopmental outcomes in relation to brain magnetic resonance imaging**.

During the last decade, structural and functional brain MRI studies in CHD fetuses, neonates before and after cardiac surgery, and adolescents have given increasing evidence of brain abnormalities in relation to factors mediating ND disabilites (Figure 2). In MRI studies, the term “cerebral white matter immaturity” has been suggested (33–35), and a rate of 20–50% of WMI in newborns prior to surgery, which is dependent on the severity of the underlying CHD, has been detected (36–39). Brain MRI studies have also shown smaller brain volumes, abnormal brain metabolism and decreases in cortical folding, and gyral development in CHD fetuses (27, 40). However, the predictive value of brain abnormalities detected *in utero* MRI on postnatal preoperative brain injury is limited. Further postnatal studies prior to cardiac surgery are needed (41). In newborns with complex CHD prior to surgery (42–46), smaller preoperative brain volumes, abnormal brain metabolism and decreases in cortical folding, and gyral development were also detected. These were associated with a poor behavioral state regulation (43). Brain maturation has been found delayed by 1 month in newborns with transposition of the great arteries or hypoplastic left heart syndrome (34). Associations between lower brain maturity at birth and increased preoperative and postoperative brain injury (47) as well as ND impairment at the age of 2 years (38) suggest that the so-called “encephalopathy of CHD” (48, 49) may increase the vulnerability of the brain to hypoxia or ischemia. This is especially true in the setting of the surgical and perioperative management, and also in terms of a longer preoperative period between birth and surgery (50). After cardiac surgery, more than 50% of the neonates provide MRI signs of WMI (36–38).

In summary, the brain immaturity and abnormality in infants with CHD seem to be a complex disturbance with destructive and developmental elements, similar to the encephalopathy first described in premature infants (51). Beginning in the fetal period, the encephalopathy of CHD is a major innate risk factor for preoperative, perioperative, and postoperative additional hypoxic or ischemic brain injury and subsequent ND impairment.

There are also important implications in long-term follow-up linking brain abnormalities in CHD to later ND delay. Brain volumes, including hippocampal volume, remain smaller into adolescence. They are accompanied by reduced ND outcomes (52, 53). MRI macrostructural brain abnormalities (54) and regions of reduced white matter microstructure (55) in TGA adolescents have been found correlated with neurocognitive decline. Diminished white matter microstructure may contribute to cognitive compromise in adolescents who underwent open-heart surgery in infancy. Recently, special brain MRI investigations have suggested that disorganization of neuronal networks may contribute to increased attention deficiency hyperactivity disease symptoms in adolescents with TGA (56).

## Conclusion

Besides physical morbidity, ND and psychosocial disabilities are the most common long-term risks of critical CHD. Surgical factors seem to be less important than innate patient and preoperative factors and postoperative events in predicting ND outcomes after cardiac surgery in infancy. Since the variance in percentage explained by the considered clinical variables is quite low, as-yet unknown intrauterine and genetic factors should be investigated. The risk of delayed brain maturation and brain injury evaluated by MRI in fetuses and in neonates with CHD prior to cardiac surgery is of importance.

While the predisposing factors for ND disorders are predominantly innate and while only a few of them are modifiable, research focuses on new approaches for neuroprotection. Examples for such research are cerebral vascular autoregulation monitoring, new perioperative brain biomarkers, and perioperative EEG monitoring. Sophisticated longitudinal brain MRI studies with systematic correlation to ND outcomes aim at an improved risk stratification and therapy for the CHD population.

## Author Contributions

HH-G is the only author of the manuscript and contributed concept, text, and reference list.

## Conflict of Interest Statement

The author declares that the research was conducted in the absence of any commercial or financial relationships that could be construed as a potential conflict of interest.
